# Notes and Notices

**Published:** 1892-06

**Authors:** 


### NOTES AND NOTICES.

SIMILIA SIMILLIBUS? "Diagnosis: Syphilis, exanthema maculopapulosum corporis, adenitis universalis, ulcus durum." Remedy, "Isobutylorthocresoliodide." Can our friends see the point?—HITCHCOCK.

REED & CARNRICK'S PREPARATIONS.—We desire to call the attention of our readers to the new advertisment of Reed & Carnrick.

This firm have spared neither labor or expense to perfect their Infant Foods in keeping qualities by sterilization, and by placing them in hermetically sealed containers. They claim that Lacto-Preparata, an all Milk Food, for young infants, and Carnrick's Food, composed of half Lacto-Preparata and half dextrinized wheat, for use after six months of age, have now practically reached perfection in keeping qualities, and they are the only Infant Foods in the market that will alone thoroughly nurse a child during the nursing period. Their Lacto-Preparata almost perfectly resembles human milk in character, composition, and taste.

Important Notice and Removal.—To avoid failure or doubtful success in use of Peroxide of Hydrogen, be sure you get Marchand's Medicinal; no substitute can replace it, statements of dealers, interested or unscrupulous parties to the contrary notwithstanding. There is great inducement to substitute in this article, for the reason that Peroxide made for bleaching and varying trade purposes costs to produce only a fraction of what Marchand's Medicinal costs, and the unscrupulous druggist or dealer pockets the difference in profit at the expense of the physician's reputation for skill and Marchand's Peroxide of Hydrogen Medicinal.

Put up in 4 oz., 8 oz., and 16 oz. bottles only, with which every careful physician should be familiar, in order to frustrate dishonest substitution and assure success in practice. Drevet Manufacturing Co.,
28 Prince Street, New York.

Electro Homœopathy.—Dr. Berridge writes in reference to this subject: "I have a splendid prover, who is proving one of Mattei's remedies, and I hope to get him to prove others later. This is the only way to overthrow his system of Electro-Homœopathy."

The Canton Surgical Chair Co. still advertise their wonderful chair in this journal. You had better look at the advertisement on page 6 and visit our editorial office, and see the chair itself.

Mr. W. P. Cleary, the New York advertising agent of The Homœopathic Physician, has removed to 294 Broadway, New York City. The editor of this journal desires to recommend him as a reliable and painstaking business man who may be trusted implicitly in encouraging and promoting advertising connection between respectable and responsible advertisers and the best medical journals in every section of the United States.

He has been the advertising agent of this journal for several years, and has given uniform satisfaction. We can therefore give him our unqualified indorsement, and from our personal experience we do not hesitate to assert that whoever employs him in furthering advertising interests will be faithfully and efficiently served; and that, too, without expense to themselves.

The commissions we pay him we consider investments well made, from which we derive the advantage we expect, besides saving the trouble and expense of frequent personal appeals for advertising.

### AN APOLOGY.

To the Editor.

Sir:—Please permit me to make an apology to one of our most honored confreres. His friends as well as himself emphatically deny the assertion I made concerning him in my notice of Dr. Ballard. I there stated that Dr. Ballard was the student of "the *late* Dr. Geo. E. Shipman." How it happened, I'm sure I cannot tell, but Dr. Shipman as well as his friends declare that he is not "late," but is still "on deck," hale and hearty and actively pursuing his profession, and therefore I have inadvertently and unintentionally done him an injustice. I trust I may be forgiven this time, and I'll try and do better next. Fraternally, Hitchcock.

